# Longitudinal Effects of Iron Deficiency Anemia and Subsequent Repletion on Blood Parameters and the Rate and Composition of Growth in Pigs

**DOI:** 10.3390/nu10050632

**Published:** 2018-05-17

**Authors:** Laura C. Knight, Ryan N. Dilger

**Affiliations:** Piglet Nutrition & Cognition Laboratory, Department of Animal Sciences, and Division of Nutritional Sciences, University of Illinois, Urbana, IL 61801, USA; knight24@illinois.edu

**Keywords:** iron deficiency, iron deficiency anemia, pig, iron repletion, pediatric nutrition

## Abstract

Iron deficiency is reported as the most common nutrient deficiency worldwide. Due to rapid growth, infants are at particular risk for developing iron deficiency, which can easily progress to iron deficiency anemia (IDA), if not treated. The aim of this study was to determine the lasting effects of an early-life iron deficiency after a period of dietary iron repletion. Forty-two intact male pigs were fed, *ad libitum*, either control (CONT, 21.3 mg Fe/L) or iron-deficient (ID 2.72 mg Fe/L) milk replacer from postnatal day (PND) 2 to 32 (phase 1). From PND 33 to 61 (phase 2), all pigs were transitioned onto a series of industry-standard, iron-adequate diets. Blood was collected weekly from PND 7 to 28, and again on PND 35 and 56, and tissues were collected at either PND 32 or PND 61. At the end of phase 1, ID pigs exhibited reduced hematocrit (Hct; *p* < 0.0001) and hemoglobin (Hb; *p* < 0.0001) compared with CONT pigs, but neither Hct (*p* = 0.5968) nor Hb (*p* = 0.6291) differed between treatment groups after dietary iron repletion at the end of phase 2. Body weight gain was reduced (*p* < 0.0001) 58% at PND 32 in ID pigs compared with CONT pigs during phase 1, and this effect remained significant at the end of phase 2 (*p* = 0.0001), with ID pigs weighing 34% less than CONT pigs at PND 61. Analysis of peripheral protein and messenger RNA (mRNA) gene expression biomarkers yielded inconclusive results, as would be expected based on previous biomarker analyses across multiple species. These findings suggest that early-life iron status negatively influences blood parameters and growth performance, with dietary iron repletion allowing for full recovery of hematological outcomes, but not growth performance.

## 1. Introduction

Early-life nutrition profoundly influences the developing neonate, with some effects that are long-lasting and irreversible, as is the case for iron. Iron is an essential micronutrient for many biological processes, yet iron deficiency is considered the most prevalent micronutrient deficiency worldwide [1]. Though iron deficiency affects individuals of all age groups, women of childbearing age and children from birth to five years of age are at increased risk for developing iron deficiency [2]. Further, infants, specifically, are at increased risk due to heightened growth trajectories early in life. Taken together, the effects of iron deficiency on the infant can be severe, especially if iron deficiency is left untreated and progresses to IDA. The extent of detrimental outcomes in response to iron deficiency are dependent on the timing and severity of deficiency experienced [3,4]. Clinically, the most significant impact of IDA are effects on red blood cells, though this is known to recover quickly with dietary iron repletion. If severe enough, IDA has also been shown to affect growth in young children [5].

A newly established indicator of iron status is hepcidin concentration [6]. Hepcidin, synthesized in the liver, has been found to regulate iron stores in the body by binding to ferroportin, effectively regulating the transport of iron out of enterocytes [6]. Specifically, the hepcidin–ferroportin complex blocks iron uptake into circulation, and inversely, low concentrations of hepcidin allow for increased iron uptake [7]. The current study utilized the young pig as a model for the human infant. The pig has been successfully used as a preclinical model for iron deficiency, due to its marked similarities in physiological responses to this particular micronutrient deficiency [8,9,10,11]. Specifically, the young pig parallels the human infant in having low iron stores at birth, low availability of iron in porcine milk, immature iron absorption pathways in early life [12], and heightened growth trajectories after birth [13]. Further, pigs have similar physiology and nutrient requirements as human infants [14], but with relatively accelerated growth rates, making them an optimal model for early-life nutrition research. Taken together, the porcine model proves to be a powerful translational model for studying the influence of early-life nutrition on physiological and developmental systems [15].

This study sought to characterize the effects of early-life IDA, before and after dietary iron repletion, on outcomes involving growth, body composition, hematology, and metabolism. Further, we aimed to establish whether reversibility with dietary iron repletion could compensate for effects resulting from early-life iron deficiency.

## 2. Materials and Methods

### 2.1. Animal Care and Use

All animal and experimental procedures were in accordance with the National Research Council Guide for the Care and Use of Laboratory Animals and approved by the University of Illinois at Urbana-Champaign Institutional Animal Care and Use Committee. Forty-two naturally-farrowed, intact male pigs were obtained from a commercial swine farm in one of two replicates, and transferred to the University of Illinois Piglet Nutrition and Cognition Laboratory (PNCL) at PND 2. Per standard agricultural protocol, pigs were provided an intramuscular injection of a prophylactic antibiotic (0.1 mL of ceftiofur crystalline free acid as Excede, Zoetis, Parsippany, NJ, USA) within 24 h of birth. Contrary to typical agricultural procedures, pigs on this study were never provided supplemental iron (i.e., injectable iron dextran), because iron is the nutrient of interest. Recent pig studies observed hippocampal transcriptome changes [16] and possible effects of iron overload [9] after iron dextran administration in the first few days of life, which further justify our decision to not provide iron dextran to any pigs. Upon arrival to PNCL on PND 2, pigs were stratified into one of two experimental diets, described below. Pigs were provided experimental milk replacer treatments from PND 2 until PND 32 or 33 (phase 1), at which point, both treatment groups were transitioned onto a common series of industry-standard diets from PND 32 or 33 until PND 61 or 62 (phase 2).

For phase 1 of this study, 42 pigs were housed individually in custom pig rearing units (87.6 cm long, 88.9 cm wide, 50.8 cm high), which were composed of three acrylic walls, one stainless steel wall, and vinyl-coated, expanded-metal flooring. This caging environment was designed for pigs to see, hear, and smell, but not touch, neighboring pigs. Pigs were allowed to physically interact with one another for approximately 15 min each day, and each pig was provided a toy for enrichment in their home-cage throughout the study. Facility lighting was maintained on a 12 h light and dark cycle from 0800 to 2000 h, with ambient temperature set at 26.6 °C for the first 21 days of the study, and gradually lowered to 22 °C during the last seven days of phase 1.

For phase 2 of this study, 20 pigs from phase 1 were transferred to the University of Illinois Veterinary Medicine Research Farm at PND 32 or 33, and housed there until the end of the study. While in this facility, pigs were housed individually in floor pens (1.5 m^2^), and the rearing environment was maintained on a 12 h light and dark cycle from 0800 to 2000, with ambient temperature set at 22 °C.

All animal and experimental procedures were in accordance with the National Research Council Guide for the Care and Use of Laboratory Animals, and approved by the University of Illinois at Urbana-Champaign Institutional Animal Care and Use Committee. Approval for this research project was confirmed on 3 March 2015 under the title Nutrition and Brain Development in Young Pigs, and was terminated on 7 February 2018.

### 2.2. Dietary Treatments

For phase 1 of this study, pigs (*n* = 21 per diet) were provided one of two milk replacer treatments with varying iron content. The CONT diet was formulated to meet all of the nutrient requirements of the growing pig and was formulated to contain 106.3 mg Fe/kg milk replacer powder. The ID diet was identical to the CONT diet, with the exception that ferrous sulfate (i.e., the predominant iron source in CONT) was removed to provide only 13.6 mg Fe/kg milk replacer powder. Additionally, both diets were formulated to contain ARA (2.08 g ARA/kg milk replacer powder) and DHA (1.04 g DHA/kg milk replacer powder). Milk replacer was reconstituted fresh daily with 200 g of milk replacer powder per 800 g water. Thus, formulated iron concentrations in reconstituted pig milk replacers were 21.3 and 2.72 mg Fe/L milk replacer for the CONT and ID treatments, respectively. All pigs were provided *ad libitum* access to liquid milk replacer treatments from PND 2 until PND 32 or 33.

**Figure 1:**
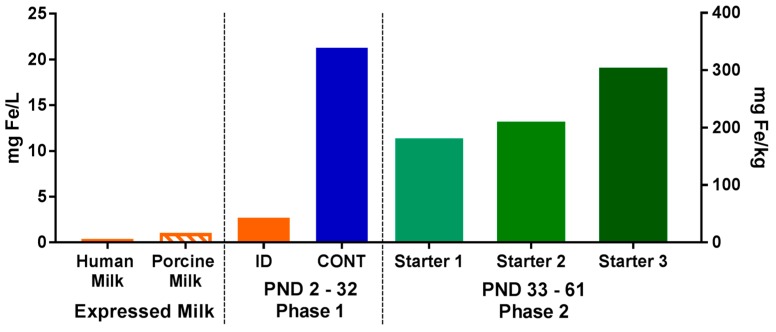
Analyzed concentrations of iron in porcine and human milks, as well as in dietary treatments during both phases of the pig study. During phase 1, pigs were fed either a control (CONT) or iron-deficient (ID) milk replacer. The CONT treatment contained 21.3 mg/L (106.3 mg/kg), and the ID treatment contained 2.72 mg/L (13.6 mg/kg). The ID treatment closely resembled the average iron content of porcine milk (*n* = 7; 1.06 mg/L) collected during a prior study [18], and is comparable to the iron concentration of human milk [7,19]. During phase 2, all pigs were fed a series of standard commercial starter diets (180–300 mg/kg). Abbreviations: CONT, control; ID, iron deficient; PND, postnatal day.

For phase 2 of this study, all pigs (*n* = 10 per diet) were transitioned onto the same common series of industry-relevant, iron-adequate diets (containing 180–300 mg Fe/kg of diet), regardless of their phase 1 dietary iron treatment group. Pigs were provided *ad libitum* access to standard complex diets (major ingredients including corn, whey, and soybean meal) and standard agricultural feeding practices were followed by sequentially switching to stage 1, 2, and 3 diets on PND 32, 41, and 50, respectively. During phase 2 of the study, all diets were formulated to meet all nutrient requirements of growing pigs [17], including iron. No zinc oxide, copper sulfate, or in-feed antibiotics were included in any diets. Analyzed values of iron in diets can be found in Figure 1.

Porcine milk was collected as part of a previous study [18]. Samples were then analyzed for mineral profiles by using standardized procedures (Mead Johnson Nutrition, Evansville, IN, USA) to establish iron content. Specifically, porcine milk samples were digested using a combination of concentrated nitric acid and 30% hydrogen peroxide at 220 °C for 10 min in a microwave digestion system (UltraWAVE; Milestone Inc., Shelton, CT, USA). After digestion, the samples were diluted to volume and quantified by inductively-coupled plasma mass spectrometry (ICP-MS; NexION 300D; Perkin Elmer, Waltham, MA, USA). The instrument was operated in kinetic energy discrimination mode using helium to reduce polyatomic interferences. All samples were analyzed in duplicate.

### 2.3. Growth Performance

Individual body weights and milk hopper weights were recorded daily, utilizing a custom-built scale (ShapeMaster Inc., Ogden, IL, USA) to assess growth performance during phase 1. During phase 2, body weights were recorded weekly utilizing a large animal scale (Osborne Industries, Osborne, KS, USA), and net disappearance of feed was recorded daily by weighing back each feeder on a validated digital scale (Pelouze Scale Corp, Oak Brook, IL, USA).

### 2.4. Hematological Outcomes

Approximately 100 µL of blood was collected from the ear of each pig on PND 7, 14, 21, 28, 35, and 56. All samples were immediately subjected to testing using a clinical blood analyzer (i-STAT; Abbott Point of Care, Princeton, NJ, USA) utilizing i-STAT Chem8+ cartridges (Abbott Point of Care, Princeton, NJ, USA). The clinical equipment passed all internal calibration and quality assurance checks prior to analysis at each sampling time-point.

### 2.5. Blood and Tissue Collection, Processing, and Analysis

At PND 32 (CONT, *n* = 6; ID, *n* = 7) and PND 61 and 62 (*n* = 20; *n* = 10 per phase 1 diet), pigs were euthanized for tissue and blood collection. All animals were euthanized in a food-deprived state, with no access to dietary treatments for at least 6 h prior to euthanasia. Pigs were anesthetized using an intramuscular injection of telazol/ketamine/xylazine administered at 0.022 mL/kg bodyweight (50.0 mg tiletamine plus 50.0 mg of zolazepam reconstituted with 2.50 mL ketamine (100 g/L) and 2.50 mL xylazine (100 g/L); Fort Dodge Animal Health, Overland Park, KS, USA). To ensure pigs were properly anesthetized prior to euthanasia, all pigs were tested for reflex via the eye blink response. Pigs were euthanized using a 390 mg/mL sodium pentobarbital solution (Patterson Veterinary Supply, Columbus, OH, USA) at 1 mL/5 kg body weight by intracardiac injection. Blood was collected from the jugular vein immediately after euthanasia on PND 32 and 61, and collected into evacuated serum and EDTA-containing tubes (Becton, Dickenson and Company, Franklin Lakes, NJ, USA). Serum was left at room temperature to clot for at least thirty minutes. Plasma was collected into EDTA tubes, gently inverted, and stored on ice for up to four hours until processing. Serum and plasma were processed by spinning blood down utilizing an Allegra 6R centrifuge (Beckman Coulter Life Sciences, Indianapolis, IN, USA), aliquoted, and stored at −80 °C. Serum was analyzed for hepcidin and ferritin concentration via validated porcine enzyme-linked immunosorbent assay (ELISA) kits (Elabscience, Hongshan, Hubei Province, China), with a detection range of 1.56–100 ng/mL and 4.69–300 ng/mL, for hepcidin and ferritin, respectively.

Duplicate aliquots of liver tissue (~0.5 g each) were collected immediately following euthanasia on PND 32 or 61, rinsed with ice-cold phosphate buffered saline (0.01 mM), snap frozen in liquid nitrogen, and stored at −80 °C until processing. Liver samples were processed and analyzed via the same validated porcine ELISA kits mentioned above for hepcidin and ferritin concentrations. Proximal duodenum scrapings were collected in duplicate, snap frozen, and stored at −80 °C until processing, to analyze divalent metal transporter 1 (DMT1) concentration via validated porcine ELISA kits (DL Develop, Wuxi, Jiangsu Province, China), with a detection range of 0.156–10 ng/mL. All measures analyzed by ELISA kit were assessed in duplicate, and run according to the manufacturer’s instructions. Samples with a result above the upper limit of the kit’s detection range were diluted and re-analyzed.

Quantitative real-time polymerase chain reaction (qRT-PCR) was utilized to quantify gene expression of ferritin and hepcidin in liver samples, and DMT1 in proximal duodenum scrapings. Frozen liver and proximal duodenum scrapings aliquots (50 to 100 mg) were placed into 2 mL microcentrifuge tubes containing a 5 mm stainless steel bead and one mL of TRIzol reagent (Invitrogen, Carlsbad, CA, USA), to enable tissue disruption for two minutes at 30 Hz (TissueLyser II, Qiagen, Valencia, CA, USA). Ribonucleic acid (RNA) extraction was carried out according to manufacturer recommendations for TRIzol reagent, and total extracted RNA was quantified using a spectrophotometer (NanoDrop ND-1000, NanoDrop Technologies, Wilmington, DE, USA). Complimentary DNA (cDNA) was transcribed from the isolated RNA using a high capacity cDNA Reverse Transcriptase kit (Thermo Fisher Scientific Inc., Waltham, MA, USA), with samples placed in a thermocycler (Bio-Rad, Hercules, CA, USA) set to run at 25 °C for 10 min, 37 °C for 120 min, 85 °C for 5 min, and then cooled at 4 °C, and held at 4 °C overnight. Samples were then removed and kept at −20 °C until plating. The TaqMan Gene Expression Assay (Thermo Fisher Scientific Inc., Waltham, MA, USA) was used to perform qRT-PCR to quantify relative gene expression of porcine target genes hepcidin, ferritin, and DMT1 (HAMP, FTH1, and SLC11A2, respectively) and the reference gene β-actin (Applied Biosystems, Carlsbad, CA, USA) [20]. Sample cDNA was amplified using TaqMan (Thermo Fisher Scientific Inc., Waltham, MA, USA) oligonucleotide probes containing 5′ fluorescent reporter dye (6-FAM) and 3′ non-fluorescent quencher dye, and fluorescence was determined using a QuantStudio 7 Flex Real-Time PCR System (Applied Biosystems, Foster City, CA, USA). To normalize gene expression, parallel amplification of endogenous β-actin was performed in triplicate for each sample. Relative gene expression was then calculated using the comparative threshold cycle method [21] and results are expressed as fold-change relative to CONT pigs.

Following sample collection procedures at the end of phase 1, nine pigs per treatment group were used to perform a carcass composition analysis at the University of Illinois Meat Science Laboratory (Urbana, IL, USA) using standardized procedures. Specifically, carcasses were skinned by hand, and an air skinner was utilized to leave subcutaneous fat with the carcass. The head, feet, testicles, and heart were then removed, leaving only muscle, fat, and associated connective tissue (i.e., total soft tissue), which was used to collect a standardized final carcass weight. All bones were separated from soft tissue and knife-scraped to remove residual tissue. Dissected carcasses were divided and weighed, with categories including skin, bone, and soft tissue. Soft tissue was prepared for proximate composition analysis by grinding and homogenizing all soft tissue through a commercial bowl chopper. A 10 g sample of soft tissue was oven-dried at 110 °C for approximately 24 h to determine percentage moisture. The dried sample was then washed multiple times in an azeotropic mixture of warm chloroform/methanol, as described by Swensen et al. [22], to determine the fat content of the soft tissue. Finally, the percentage of carcass weight, categorized as fat vs. fat-free lean, was calculated for each pig.

### 2.6. Statistical Analysis

All researchers involved in this study (i.e., those performing daily procedures, data collection, and data analysis steps) remained blind to dietary treatment identity until final data analyses had been completed. Data were analyzed using the MIXED procedure of SAS (version 9.4, SAS Institute, Cary, NC, USA). A replicate was considered to be a random variable. Hematological and bodyweight outcomes were collected from the same pig at multiple time-points, and thus, were analyzed using a 2-way repeated measures analysis of variance (ANOVA). Interactive effects were defined as an interaction between diet (CONT vs. ID) and PND. All other data were collected at a single time-point, and thus, were analyzed using a one-way ANOVA to determine the effect of phase 1 dietary iron status. Nine pigs were omitted from tissue analysis procedures due to failure to thrive or complications during neuroimaging procedures. Data were analyzed for outliers (defined as having a studentized residual with an absolute value greater than 3) and outliers were removed prior to statistical analysis. Significance was accepted at *p* ≤ 0.05, trends were defined as 0.05 < *p* < 0.10, and data are presented as least-squares means with pooled standard errors of the mean (SEM).

## 3. Results

### 3.1. Growth Performance

**Figure 2:**
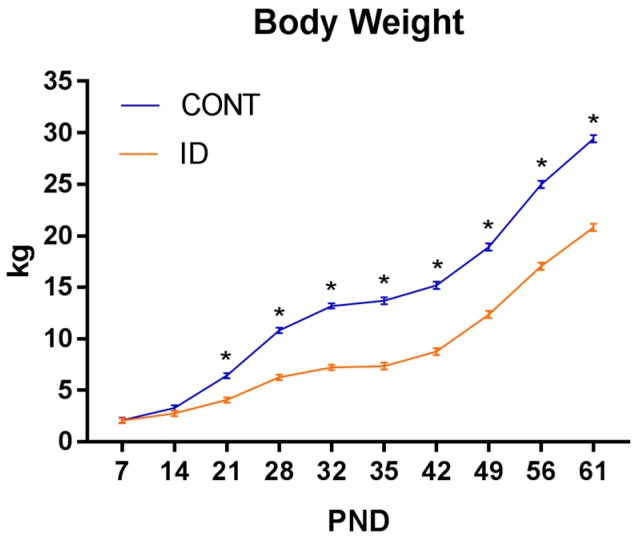
Effect of early-life iron status on pig body weight. Early-life dietary iron status has long-lasting influences on pig body weight for a time longer than the period spent in an ID state. Interactive effects of diet and PND were observed (*p* < 0.001), and SEM error bars are present. Abbreviations: CONT, control; ID, iron deficient; PND, postnatal day; SEM, standard error of the mean.

A repeated-measures ANOVA revealed an interaction effect of dietary iron status and PND (*p* < 0.001). Significant effects on body weight for diet (*p* < 0.001) and PND (*p* < 0.001) were observed, with ID pigs weighing less than CONT pigs. Separation between the CONT and ID groups on bodyweight were statistically different by PND 15 (*p* = 0.03), with ID pigs weighing less than CONT pigs from that time-point and continuously through PND 61 (Figure 2).

All growth performance data are presented in Table 1. Average daily gain (ADG, *p* < 0.001), average daily feed intake (ADFI, *p* < 0.001), and the efficiency of weight gain (i.e., gain-to-feed ratio, G/F; *p* < 0.001) were consistently lower in ID pigs during phase 1. During phase 2, ID pigs had lower ADG (*p* = 0.03) and ADFI values (*p* = 0.003), but tended to have higher G/F (*p* = 0.066). From PND 3 to 61, ADG and ADFI were lower (*p* < 0.001) for ID pigs compared to CONT pigs, but no treatment effects were observed for G/F (*p* = 0.150) from PND 3 to 61.

### 3.2. Organ Characteristics and Body Composition

**Figure 3:**
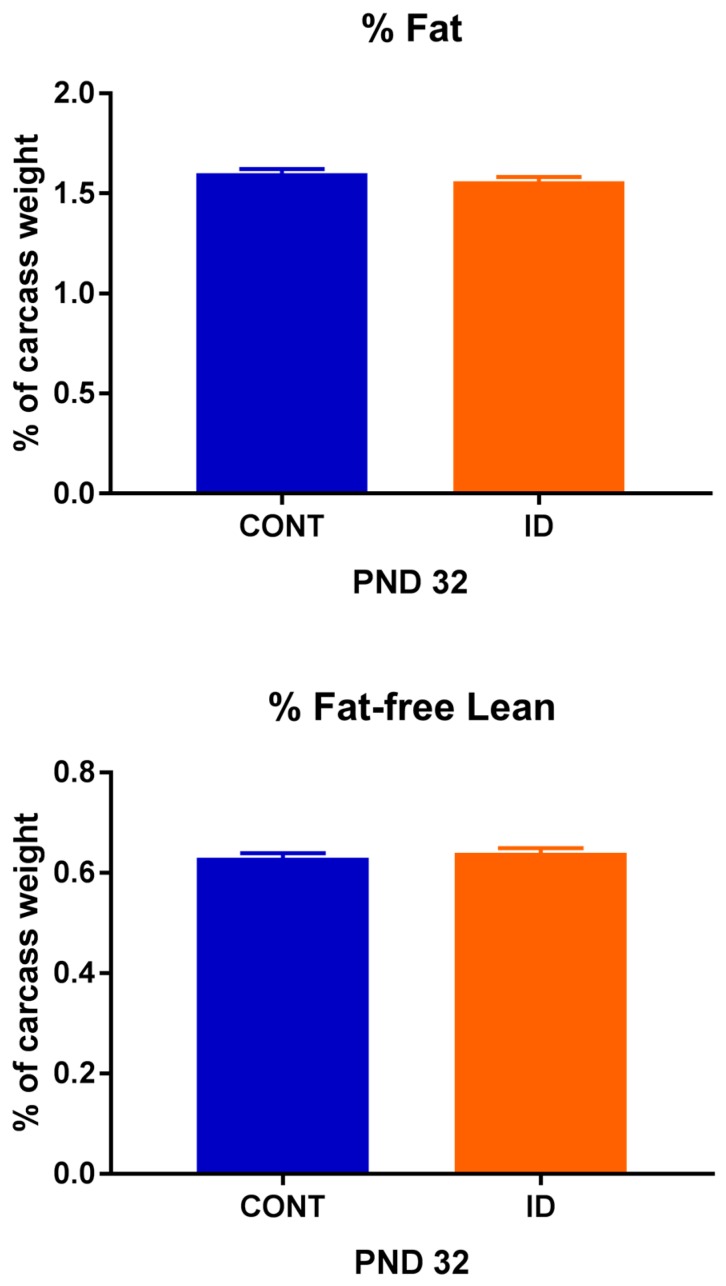
Percent fat and percent fat free lean of pigs differing in early-life iron status. Early-life iron status did not affect body composition in young pigs despite differences in overall bodyweight. No effects of dietary iron status were observed for percent carcass fat (*p* = 0.322) or percent carcass fat-free lean (*p* = 0.347). Abbreviations: CONT, control; ID, iron deficient; PND, postnatal day.

Organ weights were analyzed on an absolute basis, as well as a proportion relative to individual pig body weights, due to significant effects observed (Table 2). On an absolute basis, no differences were observed between treatment groups for brain weight at PND 32 (*p* = 0.268) or 61 (*p* = 0.136), however, ID pigs had smaller liver (*p* ≤ 0.01) and small intestine (*p* ≤ 0.01) weights at both time-points. Relative to body weight, ID pigs had a larger (*p* ≤ 0.001) proportion attributed to the brain than did CONT pigs. Further, ID pigs had higher relative liver (*p* = 0.009) and small intestine (*p* = 0.006) weights on PND 61, but not PND 32. Carcass weight (*p* = 0.001), raw bone weight (*p* = 0.002), fat weight (*p* = 0.001), and lean weight (*p* = 0.002) were all reduced in ID pigs at PND 32 (data not shown), but no differences were observed between treatment groups for percentage carcass fat (*p* = 0.322) or percentage carcass fat-free lean (*p* = 0.347) (Figure 3).

### 3.3. Hematological Outcomes

**Figure 4:**
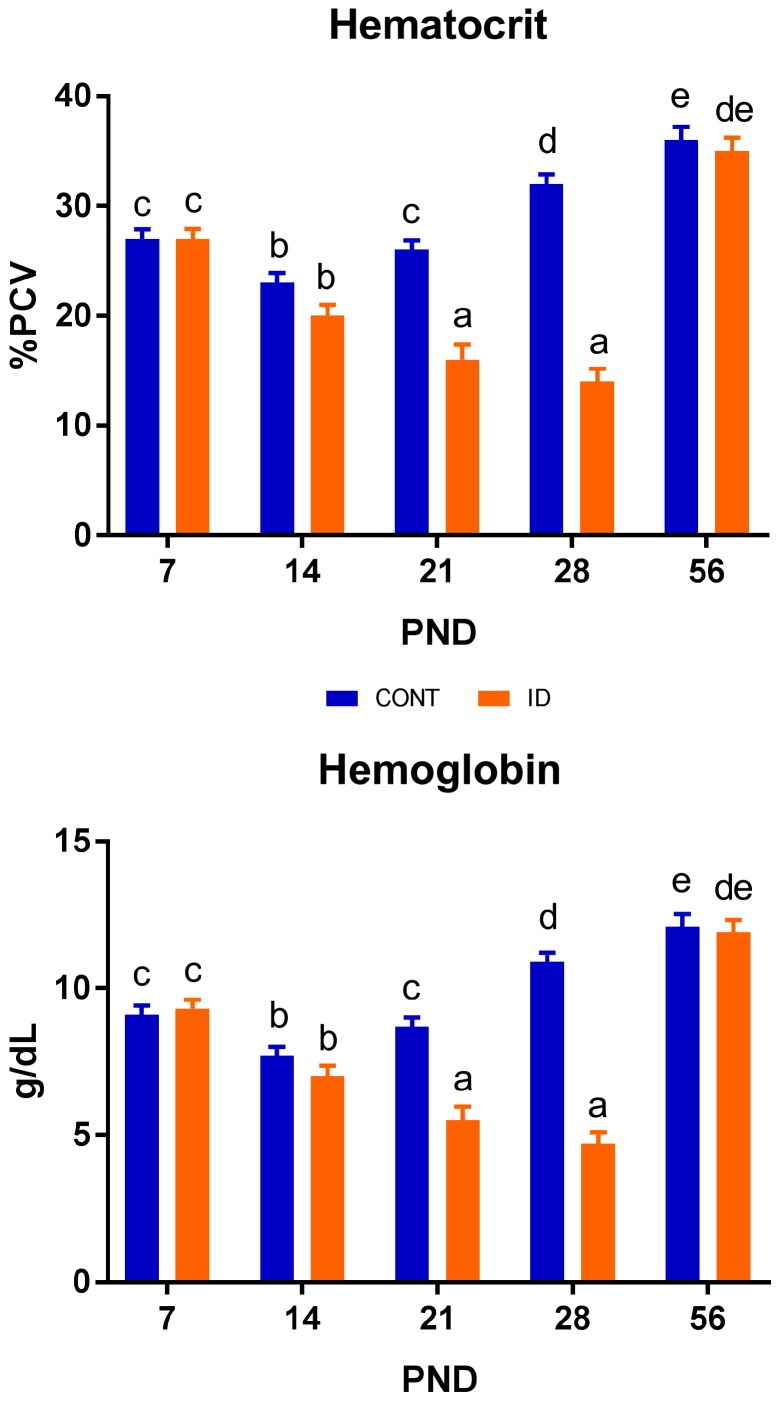
Hematocrit and hemoglobin concentrations in pigs differing in early-life iron status. Early-life iron status influences hematocrit and hemoglobin concentration in young pigs. Interactive effects of dietary iron status and PND, as well as main effects of diet and PND, were observed for hematocrit (*p* < 0.001) and hemoglobin (*p* < 0.001). ^a–f^ Means without a common superscript letter differ, *p* < 0.05. Abbreviations: CONT, control; ID, iron deficient; PND, postnatal day; % PCV, percent packed cell volume.

Time-dependent Hct and Hb data are presented in Figure 4. Interactive effects of dietary iron status and PND were observed for both Hct (*p* < 0.001) and Hb (*p* < 0.001). Effects of diet (*p* < 0.001) and PND (*p* < 0.001) were also observed in Hct and Hb. Hct and Hb values varied throughout the study. A clear separation between dietary groups was observed for both Hct and Hb by PND 21, with ID pigs having lower Hct (*p* < 0.001) and Hb (*p* < 0.001) concentrations compared with CONT pigs. This continued through the last time-point of phase 1, but this effect was fully recovered by PND 56.

Clinical blood chemistry data are presented in Table 3. Interactive effects of dietary iron status and PND (*p* ≤ 0.01) were observed for sodium and creatinine. Postnatal day had a significant main effect on all hematological outcomes (*p* ≤ 0.01). Main effects of diet were observed with sodium (*p* = 0.002), chloride (*p* = 0.041), ionized calcium (*p* < 0.001), and creatinine (*p* < 0.001) all being decreased in ID pigs compared with CONT pigs. The decrease in sodium was first observed on PND 28 (*p* < 0.001), and fully recovered by PND 56 (*p* = 0.937). Chloride was decreased (*p* = 0.028) on PND 21 only. Ionized calcium was lower (*p* = 0.012) in ID pigs at PND 14, but fully recovered by PND 56 (*p* = 0.725). The drop in creatinine values in ID versus CONT pigs was first observed at PND 21 (*p* = 0.034) and remained lower through PND 56.

### 3.4. Peripheral Biomarker Outcomes

Analysis of peripheral protein expression biomarkers revealed no differences in the following: DMT1 (*p* = 0.229) in the proximal duodenum, and hepcidin (*p* = 0.569) and ferritin (*p* = 0.446) in the liver at PND 32. Additionally, no significance was observed in DMT1 (*p* = 0.109) in the proximal duodenum, or in hepcidin (*p* = 0.515) and ferritin (*p* = 0.737) in the liver at PND 61. Analysis of serum hepcidin and ferritin were not above detectable limits with the ELISA kits that were used (data not shown).

Analysis of peripheral mRNA gene expression biomarkers displayed no difference (*p* = 0.593) in ferritin, but decreased (*p* = 0.037) hepcidin was observed in the liver of ID pigs compared with CONT pigs at PND 32. In the proximal duodenum, DMT1 was trending higher (*p* = 0.091) in ID pigs at PND 32 compared to CONT pigs. No differences were observed in ferritin (*p* = 0.920) or hepcidin (*p* = 0.199) in the liver, or in DMT1 (*p* = 0.909) in the proximal duodenum between ID and CONT pigs at PND 61. All gene expression data are reported as fold-change relative to endogenous β-actin expression of the CONT group within certain time-points (Table 4).

## 4. Discussion

This study utilized the biomedical pig model to assess the effects of early-life IDA and if recovery of hematological parameters and growth performance is possible with dietary iron repletion later in life. A novel aspect of this study is the longitudinal assessment and ability to quantify whether compensation for early-life iron deficiency was possible through dietary iron repletion. Our results indicate that although blood characteristics quickly recover, the effects of iron deficiency on various growth parameters remain evident later in life.

### 4.1. Growth Performance

Body weight did not differ between groups at the beginning of the study, and for the first two weeks of life. Once IDA was clearly established in the ID group by PND 15, ID pigs began displaying reduced body weights compared with CONT pigs; an effect that remained through study conclusion. Interestingly, these differences in body weight persisted despite dietary iron repletion occurring halfway through the trial. Feed intake was also reduced in ID pigs beginning in the second week of life, and essentially lasting through study conclusion. This is reflected in decreases in both ADG and ADFI during phase 1, phase 2, and overall, which is congruent with other studies in young pigs [9] and in human infants [23]. However, one study evaluating the effects of iron on cognition in iron-adequate, mildly-deficient, and severely-deficient young pigs did not report effects in growth performance, though this could be due to a method of feeding that was calorically restricted, based on body weight [8]. Importantly, the efficiency of body weight gain was lower in ID pigs, but only during phase 1 (i.e., when the iron deficiency was applied) and not during phase 2 (i.e., the iron repletion period). This suggests compensatory body weight gain may have occurred with iron repletion. As growth is one of the most basic and most sensitive indicators of overall metabolism and health status [24], this is of major concern for infant development. These findings contribute to the concept that iron is an essential nutrient for proper growth and development.

### 4.2. Organ Characteristics and Body Composition

On an absolute basis, brain weight did not differ at either time-point between the CONT and ID groups. This was interesting in that the body weights of the two groups were so starkly different. When analyzed on a relative basis, however, brain weight amounted to a greater proportion of body weight in ID pigs compared with CONT pigs at both PND 32 and 61. This suggests that brain growth was somewhat preserved in ID pigs, with a greater priority of nutrients being partitioned into brain development versus other organs in the body. The effects of energy partitioning have been well-studied in animal models [25] as well as in the infant [26,27]. On an absolute basis, both liver and small intestine weights were reduced in ID pigs compared to CONT pigs at both time-points. Interestingly, on a relative basis, liver and small intestine weights of ID pigs did not differ from CONT pigs at PND 32, but were a higher relative proportion of body weight than CONT pigs at PND 61. This finding suggests that after the period of early-life iron deficiency, there was a time of compensatory liver growth once iron was replete in the diet that carried out through study conclusion. Therkildsen and colleagues previously observed a similar phenomenon of compensatory growth in pigs after a time of feed restriction [28].

When analyzing body composition, it was clear that IDA had a significant effect between the two groups. Absolute carcass, raw bone, fat, lean, and bone mass weights were all decreased in ID pigs compared with CONT pigs at PND 32. This finding complements the decreased body weights in ID pigs observed throughout phase 1 of the study. It was expected that ID pigs would be leaner than CONT pigs to make up for the conservation of brain development [29,30], but interestingly, on a percent basis, fat and fat-free lean were not different between groups at PND 32. This is in contrast to Bauer and colleagues, who found that brain sparing in intrauterine growth-restricted pigs was present in conjunction with decreased muscle mass [31]. It has been well established in cases of intrauterine growth models, or animals with insufficient nutrient provision, that blood flow is redirected, such that vital organs like the brain receive increased blood flow, and blood flow to peripheral tissues, like muscle, is decreased [29,32]. It is unclear why a similar effect of decreased carcass fat-free lean (i.e., representing decreased muscle mass) was not observed in the current study, and future work should seek to elucidate this finding. To our knowledge, there do not appear to be any other studies that have assessed overall body composition outcomes in an ID pig model.

### 4.3. Hematological Outcomes

Hemoglobin is a standard measure used to evaluate iron status and diagnose anemia [2]. For pigs, a Hb concentration of 9 g/dL and above is considered adequate, and a level at which optimal performance may occur [33]. Borderline anemia is indicated at 8 g/dL, and anemia is diagnosed at 7 g/dL and below [33,34]. Hb values of 6 g/dL or less are associated with decreased growth performance and, if low enough, increased mortality [33,35]. At PND 7, all pigs on study displayed adequate Hb values, and exhibited a drop to borderline anemia by PND 14. This was expected, as Miller and colleagues reported that Hct and Hb levels typically fall approximately 25% after birth [36]. Such an effect is avoided in the swine industry as it is the standard agricultural practice to administer approximately 250 mg of iron via iron dextran im to every pig shortly after birth, to prevent anemia in nursing pigs [35]. No pigs in our study received iron dextran due to iron being the nutrient of interest, and pigs only had access to iron via the diet throughout the study. Through PND 21, CONT pigs maintained optimal Hb concentrations, while ID pigs exhibited a stark decrease in levels, characterized as severe anemia [33] by this time-point. The exceedingly low Hb values in ID pigs were reflected in decreased growth performance as Victor and colleagues [33] suggested, and these effects on growth performance were maintained even after dietary iron repletion, though Hb concentrations of ID pigs were normalized to match CONT pigs by study conclusion. Thus, while hematological outcomes may be recovered via dietary iron repletion, the detrimental effects of iron deficiency anemia on growth and development remain.

Hematocrit is another hematological test frequently used to assess iron status in conjunction with Hb [7,37]. Pigs in the current study displayed Hct values that followed a similar pattern as Hb in the ID and CONT pigs, with a distinct separation of treatments occurring on PND 21. Notably, CONT pigs exhibited increased Hct values compared with ID pigs. This carried through the end of phase 1. Ventrella et al. found that Hct levels were approximately 20% for five day old pigs, and 29% for pigs thirty days of age [13]. Our pigs displayed higher Hct levels at PND 7 compared with five-day-old, sow-reared pigs [13]. While porcine milk contains only low iron concentrations [36], milk consumption by pigs through nursing the sow likely explains the discrepancy in Hct values between studies. At PND 21 and 28, CONT pigs displayed Hct levels comparable with Ventrella and colleagues’ thirty-day-old pigs [13], while Hct values of ID pigs dropped well below this range to 14% by the end of phase 1. The observed decline in Hct of ID pigs followed a similar pattern to severely ID young pigs in another study evaluating the effects of early-life iron deficiency [8]. Similar to our study, Rytych et al. did not provide their severely ID group with an iron dextran injection [8], and they provided a diet similar in iron content to that which was provided in our study. Thus, this further establishes that our ID pigs were in a severely anemic state by PND 32. By PND 56, Hct values were recovered in ID pigs to a value not different than CONT pigs, which speaks to the effects of dietary iron repletion. Thus, it appears that hematological outcomes influenced by early-life iron deficiency are able to recover upon dietary iron repletion.

Analysis of electrolytes in the blood revealed decreased levels of creatinine, ionized calcium, sodium, and chloride in ID pigs compared to CONT pigs, with creatinine values decreased in ID pigs from PND 21 through study conclusion. IDA was clearly established in the ID group by PND 21. Further, stunted growth was seen in the current study from PND 15 to 61, with ID pigs displaying lower body weights compared with CONT pigs. It has been established that severe malnutrition and stunted growth in children can contribute to decreased creatinine levels [38]. Taken together, this could explain why ID pigs had a noticeable drop in creatinine that carried through the end of phase 2. To our knowledge, there do not appear to be any studies in the pig model evaluating normal ionized calcium concentrations in the blood. Thus, it remains unclear why lower values were observed in the ID group. However, it is well established that ionized calcium is involved in muscle contraction [39,40], and circulating ionized calcium is critical for facilitating muscle function [41]. Therefore, the lower muscle mass of the ID pigs may contribute to the lower concentration of ionized calcium as well. Decreases in sodium and chloride were observed in the ID group compared with CONT pigs. With regard to sodium, ID pig’s concentrations still fell within range of a previous study by Ventrella and colleagues looking to establish clinical reference ranges for the biomedical piglet model [13]. Sodium in the body is tightly regulated to avoid mortality [42]. Taken together, these data suggest that pigs were within an acceptable sodium range to avoid mortality, however, ID pigs may have been effected by hyponatremia. In humans, decreases in blood chloride and sodium values have been observed in cases of excess loss of body fluids, such as prolonged diarrhea [43]. It should also be noted that blood sodium concentrations are closely related to chloride levels [43], thus, the decrease in sodium could have contributed to the decrease in chloride in ID pigs during phase 1. Although urine samples were not evaluated for electrolytes, it is possible that ID pigs had greater fluid loss than CONT pigs, thus explaining the observation of decreased sodium and chloride levels. Future studies looking specifically at urine loss and diarrheal episodes in ID pigs are needed to verify this finding.

### 4.4. Peripheral Biomarker Outcomes

Despite a clear response to IDA in the ID pigs on trial, biological responses were not detected in protein expression analysis of hepcidin, DMT1, or ferritin using validated porcine-specific ELISA kits on tissues collected at PND 32. Gene expression analysis yielded no biological response of ferritin in ID pigs, though DMT1 was trending higher, and hepcidin mRNA expression was lower in ID pigs compared to CONT pigs. We expected that hepcidin concentrations, both in circulation and in the liver, would be decreased in ID pigs to allow for greater iron absorption when pigs were exhibiting the strongest signs of IDA (i.e., at the end of phase 1). Hepcidin is known to regulate iron status by binding to ferroportin in high iron environments, and therefore, decrease absorption to prevent iron overload [37]. Conversely, in cases of iron deficiency, hepcidin expression is suppressed to allow increased iron absorption [44]. Decreased gene expression of hepcidin was observed in the liver of ID pigs compared with CONT pigs. This finding is congruent with a study performed by Nicolas et al., who found decreased gene expression of hepcidin in ID mice [45]. Moreover, DMT1 expression was expected to increase in the duodenum to allow greater iron absorption [46] in ID pigs at PND 32, as this has been established as a trademark response in an attempt to counteract the onset of an ID state [47]. Further, Jiang and colleagues noted a marked increase of DMT1 mRNA expression in proximal duodenum scrapings of ID Belgrade rats, further backing this notion [48]. Gene expression suggested a trend for higher DMT1 gene expression in the ID group compared with CONT pigs. Finally, serum ferritin is currently considered a highly-sensitive biomarker for iron deficiency [49], directly correlating to the body iron stores [7,50], and it is well established that iron deficiency decreases both tissue (i.e., stored) and circulating ferritin concentrations [37]. Taken together, despite the decline in hepcidin and increase in DMT1 to allow for greater uptake of iron, levels of circulating and storage ferritin were expected to drop in the ID group at PND 32, due to the severity of IDA. The decrease in ferritin due to iron deficiency has been observed in pigs [51] and human infants [6,52]. Further, it has been well established that a decline in serum ferritin, and thus storage ferritin, occurs before a decrease in Hb is observed. This confirms the thought that a similar effect should have been observed at the PND 32 time-point in ID pigs established by Hct and Hb values decreased enough to be experiencing severe anemia. Thus, it is surprising that neither protein nor gene expression analyses yielded decreased ferritin outcomes in ID pigs compared with CONT pigs. Although colorimetric analyses were not quantified, anecdotal visual evidence confirms decreased liver iron content at PND 32 and apparent recovery in pigs assigned to the ID group as compared with CONT pigs (Supplementary Figure S1). Consequently, after dietary iron repletion, we expected ID pigs would have concomitant increases in serum ferritin and hepcidin, and decreased DMT1 concentrations to match CONT pigs by PND 61. This effect was observed in gene expression of hepcidin, with concentrations recovering to a value not different than CONT pigs. However, these findings warrant further research to establish why expected increases in DMT1, declines in ferritin, and more substantial evidence of decreases in hepcidin were not observed in gene expression. Furthermore, the amount of recovery time required to fully reverse blood and tissue indicators of early-life iron deficiency remains to be seen.

## 5. Conclusions

Our study made use of a severely-anemic pig model using only a low-iron diet. We observed stark decreases in Hct and Hb concentrations in ID pigs after the first two weeks of life and lasting through the end of phase 1, but Hct and Hb both quickly recovered to levels equivalent to CONT pigs after dietary iron repletion during phase 2. However, early-life IDA caused long-lasting effects on growth and organ characteristics in the pig. As such, ID pigs exhibited severely decreased feed intake and body weight gain compared with CONT pigs, and these effects remained even after dietary iron repletion. These findings suggest that early-life ID disrupts growth performance for a period of time longer than the amount of time spent in an anemic state. These findings confirm not only that iron is an essential nutrient for proper growth and development, but that some outcomes are responsive to iron repletion while others are not. Protein and mRNA gene expression outcomes of biomarkers known to be involved in iron uptake and storage did not provide definitive evidence in our pigs, though others have reported conclusive evidence on how iron deficiency effects these biomarkers across multiple species. The young pig proved to be an appropriate translational model to study the effects of nutrient deficiencies, specifically iron, and how deficiencies may affect development of both young pigs and human alike. Furthermore, it highlights a critical window in which iron is imperative to ensure proper growth of the neonate. Future studies are needed to evaluate whether growth can be fully recovered with a longer time spent on an iron replete diet. Moreover, future work should seek to evaluate the effects of IDA on DMT1, hepcidin, and ferritin to establish reference ranges for the pig as a biomedical subject, whether these biomarkers can quickly recover after iron repletion in the diet, and how long repletion is necessary to fully recover liver iron status.

## Figures and Tables

**Table 1:** Growth performance of pigs differing in early-life iron status ^1^.

Measure	Diet		Pooled	*p*-Value ^2^
	CONT	ID	SEM	
PND 3–33				
ADG, g/day	403	190	12.3	<0.001
ADFI, g liquid/day	1739	935	50.6	<0.001
G/F, g BW/g solids	1090	950	26.5	<0.001
PND 3–61				
ADG, g/day	476	329	15.0	<0.001
ADFI, g solids/day	1128	669	58.9	<0.001
G/F, g BW/g solids	1690	1786	58.1	0.150
PND 33–61				
ADG, g/day	544	470	22.0	0.030
ADFI, g solids/day	912	733	52.0	0.003
G/F, g BW/g solids	599	646	32.3	0.066

^1^ Data presented as mean and pooled standard error of the means (SEM) for each dietary treatment group. Abbreviations: CONT, control diet; ID, iron deficient diet; SEM, standard error of the mean; PND, postnatal day; BW, body weight; ADG, average daily gain; ADFI, average daily feed intake; G/F, gain-to-feed ratio or efficiency of body weight gain. Calculations reflect a reconstitution rate of 20% solids for milk replacer treatments administered from PND 3–33 (phase 1 of the study). ^2^ *p*-Values for the main effect of early-life dietary iron concentration.

**Table 2:** Organ characteristics of pigs differing in early-life iron status ^1^.

Measure	*n* ^2^	Diet		Pooled SEM	*p*-Value ^3^
		CONT	ID		
Absolute					
PND32					
BW, kg	19	13.18	7.22	0.363	<0.001
Brain, g	11	53.43	50.57	1.774	0.268
Liver, g	9	337	181	21.4	0.001
Small intestine, g	13	578	309	63.3	0.011
PND61					
BW, kg	10	29.4	20.8	0.89	<0.001
Brain, g	19	68.38	65.44	1.358	0.136
Liver, g	11	767	587	41.2	0.011
Small intestine, g	19	964	813	54	0.008
Relative to BW					
PND32					
Brain, % of BW	11	0.43	0.75	0.053	0.001
Liver, % of BW	9	2.88	2.77	0.195	0.672
Small intestine, % of BW	13	3.95	4.64	0.280	0.102
PND61					
Brain, % of BW	19	0.24	0.32	0.011	<0.001
Liver, % of BW	11	2.55	2.81	0.058	0.009
Small intestine, % of BW	19	3.30	3.95	0.149	0.006

^1^ Data presented as mean and pooled standard error of the means (SEM) for each dietary treatment group. No interactive effects involving time were noted, so only main effects of dietary treatment are presented. Abbreviations: CONT, control diet; ID, iron deficient diet; PND, postnatal day; SEM, standard error of the mean; BW, body weight. ^2^ Total number of observations used. ^3^ *p*-Values for the main effect of early-life dietary iron concentration.

**Table 3:** Clinical blood chemistry outcomes of pigs differing in early-life iron status ^1^.

Measure	*n* ^2^	CONT						ID						Pooled SEM	*p*-Value ^3^		
		PND						PND									
		7	14	21	28	35	56	7	14	21	28	35	56		PND	Diet	Interaction
Sodium, mmol/L	176	141 ^b^	137 ^a^	137 ^a^	140 ^b^	141 ^b^	142 ^b^	142 ^b^	137 ^a^	136 ^a^	137 ^a^	136 ^a^	142 ^b^	1.1	<0.001	0.002	0.007
Potassium, mmol/L	171	5.2	5.7	6.1	6.0	5.7	6.6	5.6	5.8	5.7	5.9	5.7	6.4	0.36	0.010	0.849	0.645
Chloride, mmol/L	177	102	101	101	102	105	102	103	98	97	100	104	103	1.9	0.002	0.041	0.347
Ionized calcium, mmol/L	175	1.24	1.17	1.25	1.28	1.25	1.40	1.22	1.09	1.18	1.20	1.15	1.38	0.081	<0.001	<0.001	0.427
Total carbon dioxide, mmol/L	174	28	27	23	24	23	27	28	28	26	23	20	28	1.3	<0.001	0.749	0.099
Glucose, mg/dL	175	103	111	127	128	124	117	103	109	114	119	112	127	5.6	<0.001	0.070	0.073
Urea nitrogen, mg/dL	145	23	10	7	12	7	6	22	11	6	9	12	6	3.5	<0.001	0.955	0.747
Creatinine, mg/dL	176	0.5 ^a^	0.6 ^a^	0.7 ^b^	0.8 ^c^	1.3 ^e^	1.0 ^d^	0.6 ^a^	0.5 ^a^	0.6 ^a^	0.7 ^b^	1.1 ^d^	0.8 ^bc^	0.05	<0.001	<0.001	0.010

^1^ Data presented as mean and pooled standard error of the means (SEM) for each dietary treatment group. Main effects of dietary treatment (Diet; CONT vs. ID) and PND, and the interaction between Diet and PND are presented. ^a–e^ Means in a row without a common superscript letter differ, *p* < 0.05. Abbreviations: CONT, control diet; ID, iron deficient diet; PND, postnatal day; SEM, standard error of the mean. ^2^ Total number of observations used. ^3^ *p*-Values for the main and interactive effects of early-life dietary iron concentration.

**Table 4:** Iron-related gene expression relative from pigs differing in early-life iron status ^1^.

Measure	*n* ^2^	Diet		Pooled SEM	*p*-Value ^3^
		CONT	ID		
PND 32					
Proximal Duodenum					
DMT1	9	1.00	2.37	0.508	0.091
Liver					
Hepcidin	11	1.00	0.27	0.497	0.037
Ferritin	11	1.00	0.81	0.256	0.593
PND 61					
Proximal Duodenum					
DMT1	14	1.00	1.08	0.545	0.909
Liver					
Hepcidin	18	1.00	0.66	0.181	0.199
Ferritin	18	1.00	1.02	0.133	0.920

^1^ Gene expression values calculated as fold-change relative to endogenous expression of β-actin of control pigs within each time-point. Data presented as mean and pooled standard error of the means for each dietary treatment group. Main effects of dietary treatment (Diet; CONT vs. ID) are presented. Abbreviations: CONT, control diet; ID, iron deficient diet; PND, postnatal day; SEM, standard error of the mean; DMT1, divalent metal transporter. ^2^ Total number of observations used. ^3^ *p*-values for the main effect of early-life dietary iron concentration.

## Supplementary Materials

The following are available online at http://www.mdpi.com/2072-6643/10/5/632/s1.

## References

[1] WHO (2017). Micronutrient Deficiencies. http://www.who.int/nutrition/topics/ida/en/.

[2] McLean E., Cogswell M., Egli I., Wojdyla D., de Benoist B. (2009). Worldwide prevalence of anaemia, WHO vitamin and mineral nutrition information system, 1993–2005. Public Health Nutr..

[3] Antonides A., van Laarhoven S., van der Staay F.J., Nordquist R.E. (2016). Non-anemic iron deficiency from birth to weaning does not impair growth or memory in pigs. Front. Behav. Neurosci..

[4] Georgieff M.K. (2012). Long-term brain and behavioral consequences of early iron deficiency. Nutr. Rev..

[5] Soliman A.T., Al dabbagh M.M., Habboub A.H., Adel A., Humaidy N.A., Abushahin A. (2009). Linear growth in children with iron deficiency anemia before and after treatment. J. Trop. Pediatr..

[6] Berglund S., Lonnerdal B., Westrup B., Domellof M. (2011). Effects of iron supplementation on serum hepcidin and serum erythropoietin in low-birth-weight infants. Am. J. Clin. Nutr..

[7] Hernell O., Fewtrell M.S., Georgieff M.K., Krebs N.F., Lönnerdal B. (2015). Summary of current recommendations on iron provision and monitoring of iron status for breastfed and formula-fed infants in resource-rich and resource-constrained countries. J. Pediatr..

[8] Rytych J.L., Elmore M.R.P.P., Burton M.D., Conrad M.S., Donovan S.M., Dilger R.N., Johnson R.W. (2012). Early life iron deficiency impairs spatial cognition in neonatal piglets. J. Nutr..

[9] Antonides A., Schoonderwoerd A.C., Scholz G., Berg B.M., Nordquist R.E., van der Staay F.J. (2015). Pre-weaning dietary iron deficiency impairs spatial learning and memory in the cognitive holeboard task in piglets. Front. Behav. Neurosci..

[10] Leyshon B.J., Radlowski E.C., Mudd A.T., Steelman A.J., Johnson R.W. (2016). Postnatal iron deficiency impairs brain development in piglets. J. Nutr..

[11] Nelissen E., De Vry J., Antonides A., Paes D., Schepers M., van der Staay F.J., Prickaerts J., Vanmierlo T. (2017). Early-postnatal iron deficiency impacts plasticity in the dorsal and ventral hippocampus in piglets. Int. J. Dev. Neurosci..

[12] Starzyński R.R., Laarakkers C.M.M., Tjalsma H., Swinkels D.W., Pieszka M., Styś A., Mickiewicz M., Lipiński P. (2013). Iron supplementation in suckling piglets: How to correct iron deficiency Anemia without affecting plasma hepcidin levels. PLoS ONE.

[13] Ventrella D., Dondi F., Barone F., Serafini F., Elmi A., Giunti M., Romagnoli N., Forni M., Bacci M.L. (2016). The biomedical piglet: Establishing reference intervals for haematology and clinical chemistry parameters of two age groups with and without iron supplementation. BMC Vet. Res..

[14] Moughan P.J., Birtles M.J., Cranwell P.D., Smith W.C., Pedraza M. (1992). The piglet as a model animal for studying aspects of digestion and absorption in milk-fed human infants. World Rev. Nutr. Diet..

[15] Odle J., Lin X., Jacobi S.K., Kim S.W., Stahl C.H. (2014). The suckling piglet as an agrimedical model for the study of pediatric nutrition and metabolism. Annu. Rev. Anim. Biosci..

[16] Gan L., Yang B., Mei H. (2017). The effect of iron dextran on the transcriptome of pig hippocampus. Genes Genom..

[17] National Research Council (2012). Nutrient Requirements of Swine.

[18] Mudd A.T., Alexander L.S., Johnson S.K., Getty C.M., Malysheva O.V., Caudill M.A., Dilger R.N. (2016). Perinatal dietary choline deficiency in sows influences concentrations of choline metabolites, fatty acids, and amino acids in milk throughout lactation. J. Nutr..

[19] Friel J.K. (2017). There is no iron in human milk. J. Pediatr. Gastroenterol. Nutr..

[20] Thellin O., Zorzi W., Lakaye B., De Borman B., Coumans B., Hennen G., Grisar T., Igout A., Heinen E. (1999). Housekeeping genes as internal standards: Use and limits. J. Biotechnol..

[21] Livak K.J., Schmittgen T.D. (2001). Analysis of relative gene expression data using real-time quantitative PCR and the 2^−ΔΔ*C*T^ method. Methods.

[22] Swensen K., Ellis M., Brewer M.S., Novakofski J., McKeith F.K. (1998). Pork carcass composition: II. Use of indicator cuts for predicting carcass composition. J. Anim. Sci..

[23] Branca F., Ferrari M. (2002). Impact of micronutrient deficiencies on growth: The stunting syndrome. Ann. Nutr. Metab..

[24] Gelander L. (2006). Children’s growth: A health indicator and a diagnostic tool. Acta Paediatr. Int. J. Paediatr..

[25] Webster A.J. (1993). Energy partitioning, tissue growth and appetite control. Proc. Nutr. Soc..

[26] Peebles D.M. (2004). Fetal consequences of chronic substrate deprivation. Semin. Fetal Neonatal Med..

[27] Malamitsi-Puchner A., Nikolaou K.E., Puchner K.P. (2006). Intrauterine growth restriction, brain-sparing effect, and neurotrophins. Ann. N. Y. Acad. Sci..

[28] Therkildsen M., Riis B., Karlsson A., Kristensen L., Ertbjerg P., Purslow P.P., Oksbjerg N. (2002). Compensatory growth response in pigs, muscle protein turn-over and meat texture: Effects of restriction/realimentation period. Anim. Sci..

[29] Prado E.L., Dewey K.G. (1992). Nutrition and brain development in early life. Nutr. Rev..

[30] Eiby Y.A., Wright L.L., Kalanjati V.P., Miller S.M., Bjorkman S.T., Keates H.L., Lumbers E.R., Colditz P.B., Lingwood B.E. (2013). A pig model of the preterm neonate: Anthropometric and physiological characteristics. PLoS ONE.

[31] Bauer R., Walter B., Hoppe A., Gaser E., Lampe V., Kauf E., Zwiener U. (1998). Body weight distribution and organ size in newborn swine (*sus scrofa domestica*)—A study describing an animal model for asymmetrical intrauterine growth retardation. Exp. Toxicol. Pathol..

[32] Morrison J.L. (2008). Sheep models of intrauterine growth restriction: Fetal adaptations and consequences. Clin. Exp. Pharmacol. Physiol..

[33] Victor I., Mary I., Adv J.V. (2012). Iron nutrition and anaemia in piglets: A review. J. Vet. Adv..

[34] Lee S.H., Shinde P., Choi J., Park M., Ohh S., Kwon I.K., Pak S.I., Chae B.J. (2008). Effects of dietary iron levels on growth performance, hematological status, liver mineral concentration, fecal microflora, and diarrhea incidence in weanling pigs. Biol. Trace Elem. Res..

[35] Godyń D., Pieszka M., Lipiński P., Starzyński R.R. (2016). Diagnostics of iron deficiency anaemia in piglets in the early postnatal period—A review. Anim. Sci. Pap Rep..

[36] Miller E.R., Ullrey D.E. (1987). The pig as a model for human nutrition. Annu. Rev. Nutr..

[37] Hagemann T.M., Lewis T.V. (2010). Iron Deficiency and Overload.

[38] Hari P., Bagga A., Mahajan P., Lakshmy R. (2007). Effect of malnutrition on serum creatinine and cystatin C levels. Pediatr. Nephrol..

[39] Ruegg J.C. (1992). Calcium in Muscle Contraction.

[40] Portzehl H., Caldwell P.C., Ruegg J.C. (1964). The dependence of contraction and relaxation of muscle fibres from the crab Maia squinado on the internal concentration of free calcium ions. Biochim. Biophysica Acta.

[41] Del Valle H.B., Yaktine A.L., Taylor C.L., Ross A.C. (2011). Dietary Reference Intakes for Calcium and Vitamin D.

[42] Metheny N.M. (2011). Fluid and Electrolyte Balance.

[43] Dao C.N., Peters P.J., Kiarie J.N., Zulu I., Muiruri P., Ong’ech J., Mutsotso W., Potter D., Njobvu L., Stringer J.S.A. (2011). Hyponatremia, hypochloremia, and hypoalbuminemia predict an increased risk of mortality during the first year of antiretroviral therapy among HIV-infected Zambian and Kenyan women. AIDS Res. Hum. Retroviruses.

[44] Leong W.-I., Lönnerdal B. (2004). Hepcidin, the recently identified peptide that appears to regulate iron absorption. J. Nutr..

[45] Nicolas G., Chauvet C., Viatte L., Danan J.L., Bigard X., Devaux I., Beaumont C., Kahn A., Vaulont S. (2002). The gene encoding the iron regulatory peptide hepcidin is regulated by anemia, hypoxia, and inflammation. J. Clin. Investig..

[46] Lonnerdal B., Georgieff M.K., Hernell O. (2015). Developmental physiology of iron absorption, homeostasis and metabolism in the healthy term infant. J. Pediatr..

[47] Andrews N.C. (1999). The iron transporter DMT1. Int. J. Biochem. Cell Biol..

[48] Jiang L., Garrick M.D., Garrick L.M., Zhao L., Collins J.F. (2013). Divalent metal transporter 1 (Dmt1) mediates copper transport in the duodenum of iron-deficient rats and when overexpressed in iron-deprived HEK-293 cells. J. Nutr..

[49] Goddard A.F., James M.W., McIntyre A.S., Scott B.B. (2011). Guidelines for the management of iron deficiency anaemia. Gut.

[50] National Research Council (2000). Dietary Reference Intakes for Vitamin A, Vitamin K, Arsenic, Boron, Chromium, Copper, Iodine, Iron, Manganese, Molybdenum, Nickel, Silicon, Vanadium, and Zinc.

[51] Antileo R., Figueroa J., Valenzuela C. (2016). Characterization of a novel encapsulated oral iron supplement to prevent iron deficiency anemia in neonatal piglets. J. Anim. Sci..

[52] Van De Lagemaat M., Amesz E.M., Schaafsma A., Lafeber H.N. (2014). Iron deficiency and anemia in iron-fortified formula and human milk-fed preterm infants until 6 months post-term. Eur. J. Nutr..

