# Supplementary material for: Longitudinal Effects of Iron Deficiency Anemia and Subsequent Repletion on Blood Parameters and the Rate and Composition of Growth in Pigs
Source: Nutrients. 2018 May 17;10(5):632. doi: 10.3390/nu10050632 (PMC5986511; doi:10.3390/nu10050632)
Supplement: Supplementary file 1 [file nutrients-10-00632-s001.zip › nutrients-301884-supplementary.docx]

**Supplementary Material:**


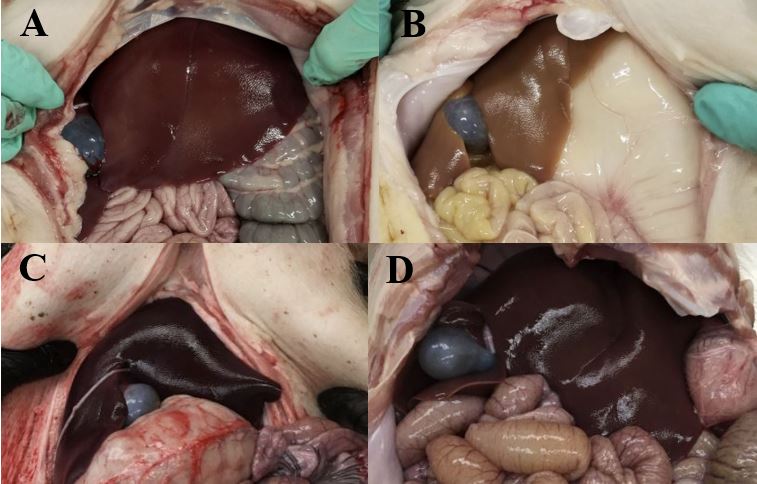


**Figure S1.** Visual evidence of decreased liver iron content in pigs differing in early-life iron status. (**A**) CONT at PND 32; (**B**) ID at PND 32; (**C**) CONT at PND 61; (**D**) ID at PND 61. Abbreviations: CONT, control; ID, iron deficient; PND, postnatal day.
